# Photothermal Ablation of Cancer Cells by Albumin-Modified Gold Nanorods and Activation of Dendritic Cells

**DOI:** 10.3390/ma12010031

**Published:** 2018-12-21

**Authors:** Xiuhui Wang, Jingchao Li, Naoki Kawazoe, Guoping Chen

**Affiliations:** 1 Research Center for Functional Materials, National Institute for Materials Science, 1-1 Namiki, Tsukuba, Ibaraki 305-0044, Japan; WANG.Xiuhui@nims.go.jp (X.W.); lijingchao1018@126.com (J.L.); KAWAZOE.Naoki@nims.go.jp (N.K.); 2 Department of Materials Science and Engineering, Graduate School of Pure and Applied Sciences, University of Tsukuba, 1-1-1 Tennodai, Tsukuba, Ibaraki 305-8571, Japan

**Keywords:** gold nanorods, cellular uptake, photothermal therapy, photothermal ablation, immune responses, cancer therapy

## Abstract

Nanoparticle-mediated photothermal therapy has been widely studied for cancer treatment. It is important to disclose how photothermally ablated tumor cells trigger immune responses. In this study, bovine serum albumin (BSA)-coated gold nanorods (BSA-coated AuNRs) were prepared and used for photothermal ablation of breast tumor cells. The BSA-coated AuNRs showed high photothermal conversion efficiency and good photothermal ablation effect towards tumor cells. The ablated tumor cells were co-cultured with immature dendritic cells (DCs) through a direct cell contacting model and diffusion model to confirm the stimulatory effects of cell–cell interaction and soluble factors released from ablated tumor cells. The results indicated that photothermally ablated tumor cells induced immune-stimulatory responses of DCs through both cell–cell interaction and soluble factors. The results should be useful for synergistic photothermal-immunotherapy of primary and metastatic cancer.

## 1. Introduction

Cancer is a severe threat to human life and causes more than seven million deaths each year [1]. Despite some technical breakthroughs that have been explored in cancer therapy, a reliable cure is still limited to such conventional methods as surgery, chemotherapy and radiotherapy [2,3]. In recent years, photothermal ablation therapy, which employs photothermal conversion agents to absorb near infrared (NIR) light and converts it into heat to ablate cancer cells, provides a highly efficient antitumor strategy for cancer therapy [4,5]. Various nanomaterials such as polypyrrole, polydopamine, indocyanine green, carbon nanotubes, graphene oxide, molybdenum sulfide, tungsten oxide, CuS nanoparticles and gold-based nanoparticles can be used for photothermal ablation therapy [6,7,8,9,10,11,12,13,14,15,16]. Among them, gold nanorods (AuNRs) have been very frequently used because of their facile large-scale preparation, tunable plasmon band and high photothermal conversion efficiency. However, nanoparticle-mediated photothermal ablation therapy, in currently used forms, has been primarily used in local cancer therapy [17,18,19].

An ideal photothermal ablation therapy should not only eradicate the primary tumor but also induce systemic anti-tumor immunity through activating the immune system to control metastatic tumor and to prevent tumor recurrence [20,21]. It has been reported that dead tumor cells and their debris can trigger potent immune responses [22,23,24]. Necrotic tumor cells can release intracellular antigens and damage-associated molecular patterns (DAMPs) such as heat shock proteins (HSPs) and high mobility group box 1 (HMGB1) from cytosol and nucleus, which can stimulate systemic immune responses [22,25,26]. Apoptotic tumor cells associated with “danger signals” such as heat stress can up-regulate expression of membrane HSPs (HSP72 and HSP60) and further elicit tumor-specific immunity [27,28,29].

During immune responses, dendritic cells (DCs) have the most effective antigen-presentation for initiation of T cell-dependent immune responses as compared to other antigen-presenting cells such as macrophages or B cells [30,31,32]. Dendritic cell-based immunotherapy caused by necrotic tumor cells and apoptotic tumor cells with “danger signals” is a promising strategy to inhibit metastatic tumor recurrence [33,34]. However, it is not clear how the immune responses of DCs can be triggered by photothermally ablated tumor cells.

In this study, BSA-coated AuNRs were prepared and used for photothermal ablation of breast tumor cells. The immune-stimulatory responses of immature DCs triggered by the photothermally ablated tumor cells were investigated by direct co-culture or transwell co-culture of immature DCs and photothermally ablated tumor cells to disclose the involvement of cell–cell interaction and soluble factors in the immune responses of DCs.

## 2. Materials and Methods

### 2.1. Preparation and Characterization of BSA-Coated AuNRs

Gold nanorods were prepared by a seed-mediated growth method [35,36,37]. First, CTAB-capped Au seeds were prepared via chemical reduction of HAuCl_4_ (0.01 mol/L, 0.25 mL) with NaBH_4_ (0.01 mol/L, 0.6 mL) in hexadecyltrimethylammonium bromide (CTAB, 0.1 mol/L, 7.5 mL) aqueous solution. The Au seeds were used within 2 h after preparation. Subsequently, a growth solution was prepared by adding HAuCl_4_ (0.01 mol/L, 15.0 mL), HCl (1 mol/L, 6.0 mL), AgNO_3_ (0.01 mol/L, 3.3 mL) and ascorbic acid (0.1 mol/L, 2.4 mL) into CTAB (0.1 mol/L, 300.0 mL) aqueous solution. Finally, 0.72 mL of Au seed solution was added to the growth solution under gentle stirring. The mixture solution was incubated under an undisturbed condition for 12 h to allow the formation of AuNRs. The AuNRs were collected by centrifugation and washed with ultrapure water to remove the free CTAB. To coat bovine serum albumin (BSA) on the surface of AuNRs, the AuNRs were dispersed in 10 mg/mL BSA aqueous solution and the solution was kept stirring at room temperature for 24 h. The BSA-coated AuNRs were collected by centrifugation and washed with ultrapure water twice and re-suspended in ultrapure water for the following experiments.

The morphology of AuNRs and BSA-coated AuNRs was characterized with a transmission electron microscope (TEM, JEM-2100F, JEOL Ltd., Tsukuba, Japan). Copper grid with super ultrahigh resolution carbon support film (Okenshoji Co., Ltd., Tokyo, Japan) was used to observe the BSA coating layer on the surface of AuNRs. Their hydrodynamic size and zeta potential were measured with a zeta-potential and particle size analyzer (ELSZ-2000, Otsuka Electronics Co., Ltd., Osaka, Japan) [38]. Three samples were used for each measurement to calculate the average and standard deviation. Fourier transform infrared (FTIR) spectrum was measured with an 8400S FTIR spectrometer (Shimadzu Corp., Kyoto, Japan). Visible-near infrared (VIS-NIR) spectrum was measured with a V-660 UV-VIS spectrophotometer (Jasco Corp., Tokyo, Japan). The concentration of gold element was determined using a Leeman Prodigy inductively coupled plasma-optical emission spectroscopy (ICP-OES, SPS3520UV-DD) system (SII Nano Technology Inc., Chiba, Japan).

### 2.2. Photothermal Performance of BSA-Coated AuNRs

To evaluate photothermal performance of BSA-coated AuNRs, 80 μL aqueous suspension of BSA-coated AuNRs in medium with different gold concentration (0.0, 0.2, 0.4, 0.6, 0.8, 1.0 mM) in quartz cuvettes at room temperature were irradiated by using an 805 nm laser (Thorlabs Inc., Newton, NJ, USA) with different power intensities of 1.4, 1.6 and 1.8 W cm^−2^ for 10 min. Irradiation at the condition of power intensity of 1.6 W cm^−2^ with irradiation time of 10 min was repeated five times to check the stability of the photothermal conversion of BSA-coated AuNRs. During laser irradiation, a digital thermometer (AS ONE Corp., Tokyo, Japan) equipped with a thermocouple probe was used to record the real-time temperature of AuNRs solution every 10 s.

### 2.3. Viability Analysis of Cells Cultured with BSA-Coated AuNRs

Viability of cells cultured with BSA-coated AuNRs was evaluated by WST-1 assay. Mouse breast tumor cells that stably express luciferase (4T1-Luc) were obtained from Japanese collection of research bioresources cell bank (JCRB, Tsukuba, Japan). The 4T1-Luc cells were subcultured in RPMI1640 medium (Gibco, Grand Island, NY, USA) supplemented with 10% FBS under 37 °C and 5% CO_2_ in a humidified incubator. The subcultured cells were harvested using a 0.25% trypsin-EDTA solution. The harvested 4T1-Luc cells were re-suspended in RPMI1640 medium to prepare cell suspension solution at a concentration of 2.0 × 10^5^ cells/mL. Subsequently, 100 μL cell suspension solution was added into each well of a 96-well plate and cultured for 12 h to allow cell adhesion. Afterwards, the medium was changed to 100 μL fresh medium containing BSA-coated AuNRs at different Au concentrations (0.0, 0.2, 0.4, 0.6, 0.8 and 1.0 mM). After being cultured for 24 h, the medium was replaced with 110 μL WST-1 dilute solution (10 μL WST-1 stock solution diluted with 100 μL medium) and the cells were cultured for another 3 h. Finally, the absorbance of WST-1 solution in each well was measured at 440 nm by a microplate reader (Benchmark Plus, Bio-Rad, Hercules, CA, USA), which was used to determine cell viability. Mean and standard deviation were calculated by performing three parallel wells for each group.

### 2.4. Cellular Uptake Assay of BSA-Coated AuNRs

In vitro cellular uptake amount of AuNRs was measured by using ICP-OES. At first, 4T1-Luc cells were seeded into 6-well plates at a density of 2 × 10^5^ cells/well and cultured for 12 h to allow cell adhesion. Subsequently, the cells were incubated with fresh medium containing BSA-coated AuNRs at different Au concentrations (0.0, 0.2, 0.4 and 0.6 mM) for 6 h. The culture medium was discarded and cells were washed with PBS 3 times to remove free BSA-coated AuNRs in medium. The cells were then detached by treating with trypsin-EDTA solution, collected by centrifugation (1000 rpm, 5 min) and re-suspended in 1.1 mL PBS. Cell concentration was measured by a hemocytometer. Finally, the remaining cells were lysed with 1.0 mL aqua regia solution (nitric acid/hydrochloric acid, *v*/*v* = 1:3) for 3 days. After being diluted with water 5 times, the Au content of each sample was determined with ICP-OES. Mean and standard deviation were calculated by performing three parallel samples for each group.

### 2.5. Photothermal Ablation of Breast Tumor Cells by BSA-Coated AuNRs

The 4T1-Luc cells (2 × 10^4^ cells/well) were seeded into 96-well plates and cultured for 12 h to allow cell adhesion. The medium was changed to 100 μL fresh medium containing BSA-coated AuNRs at different Au concentrations (0.0 mM, 0.2 mM, 0.4 mM, 0.6 mM) and the cells were cultured for 6 h. Afterwards, the medium was discarded, and the cells were washed with warm PBS 3 times to remove free BSA-coated AuNRs in medium. The 4T1-Luc cells were irradiated with an 805 nm laser at a power intensity of 1.6 W cm^−2^ for 10 min. After laser irradiation, the cells were cultured in RPMI1640 medium supplemented with 10% FBS for another 4 h. Calcein-AM/PI double staining kit (Dojindo, Kumamoto, Japan) was used to stain live and dead cells before and after laser irradiation. The stained cells in 96-well plate were observed by an inverted fluorescence microscope (Olympus, Tokyo, Japan). In addition, cell viability before and after laser irradiation was quantified by a WST-1 assay. Mean and standard deviation were calculated by performing three parallel samples for each group.

### 2.6. Interaction between DCs and Ablated Breast Tumor Cells

The 4T1-Luc cells (2 × 10^4^ cells/well) were seeded into 96-well plates and cultured for 12 h. The culture medium was removed and 100 μL fresh medium containing 0.4 mM BSA-coated AuNRs was added in each well. The cells were cultured for another 6 h. After that, the culture medium was removed and the cells were washed with PBS for 3 times. The cells in each well were irradiated with an 805 nm laser at a power intensity of 1.6 W cm^−2^ for 10 min.

Mouse bone marrow-derived immature DCs (ATCC, Manassas, VA, USA) were used to investigate immune responses induced by photothermally ablated tumor cells. Dendritic cells were cultured in MEM-α supplemented with 20% FBS and 5 ng/mL GM-CSF, whose growth property was a mixture of adherent and floating cells. For subculture of DCs, the adherent cells were detached using a 0.25% trypsin-EDTA solution, and the floating cells were directly transferred into tubes. After the detached cells and floating cells were mixed in tubes, the tubes were centrifuged at 1000 rpm for 10 min. The cells were re-suspended in fresh medium and used for subculture. Culture medium was changed once per week.

To investigate interaction between DCs and photothermally ablated 4T1-Luc cells, three experiments were conducted. The first experiment was designed to investigate the effect of soluble factors released from the ablated 4T1-Luc cells. Dendritic cells (1 × 10^4^ cells/well) were seeded in a 96-well transwell insert with a membrane pore size of 1.0 μm. The transwell insert was placed in the upper of a 96-well receiver plate containing the ablated 4T1-Luc cells, which enabled diffusion of soluble factors while blocking direct cell-to-cell contact (diffusion model). The second experiment was designed to investigate the effect of cell–cell interaction. The ablated tumor cells were carefully washed with PBS 3 times to remove the soluble factors released from the ablated 4T1-Luc cells. After washing, DCs (1 × 10^4^ cells/well) were seeded in the 96-well plate containing the ablated 4T1-Luc cells (cell contacting model). The third experiment was designed to investigate the effects of both soluble factors and cell–cell interaction. Dendritic cells (1 × 10^4^ cells/well) were directly seeded in the 96-well plate after photothermal ablation of 4T1-Luc cells (combination effects of diffusion model and cell contacting model). As positive and negative controls, DCs (1 × 10^4^ cells/well) were seeded in blank 96-well plate and cultured with or without 1 μg/mL lipopolysaccharide (LPS) stimulation, respectively. For all the experiments, DCs were cultured in MEM-α medium with 5 ng/mL GM-CSF for 24 h. Finally, DCs supernatants were harvested to measure the secretion amount of interleukin 6 (IL-6), interleukin 12 (IL-12), interleukin 1β (IL-1β) and tumor necrosis factor alpha (TNF-α) by an enzyme-linked immunosorbent assay (ELISA) kit according to the manufacturer’s instructions (PEPROTECH, Rocky Hill, NJ, USA). Mean and standard deviation were calculated by performing three parallel samples for each group.

### 2.7. Statistical Analysis

All quantitative experiments were repeated in triplicate (n = 3) and the results were reported as mean ± standard deviation (SD). One-way ANOVA statistical analysis was performed to evaluate the significance of the experimental data. A *p* value of 0.05 was set as the level of significance and the data were classified according to their *p* values and denoted by (*) for *p* < 0.05, (**) for *p* < 0.01 and (***) for *p* < 0.001.

## 3. Results

### 3.1. Preparation and Characterization of BSA-Coated AuNRs

**Figure 1:**
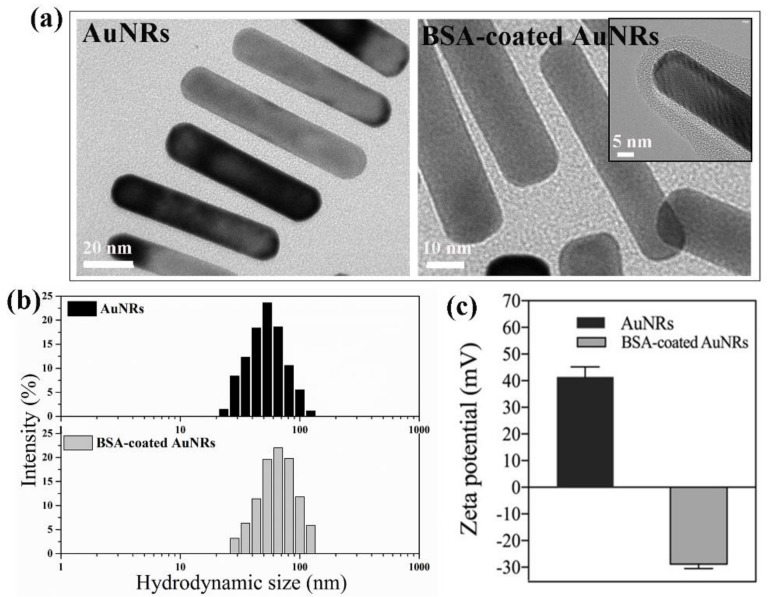
High-resolution transmission electron microscopy images (**a**), hydrodynamic size distribution (**b**) and zeta potential (**c**) of gold nanorods (AuNRs) and BSA-coated AuNRs.

The morphology of AuNRs and BSA-coated AuNRs was observed by TEM. Transmission electron microscopy images (Figure 1a) showed that the gold nanoparticles had a rod-like structure. Gold nanorods and BSA-coated AuNRs had the same morphology and dimension. Their dimension measured from TEM images was 62.3 ± 4.5 nm × 11.6 ± 3.8 nm. The TEM image of BSA-coated AuNRs demonstrated the BSA coating layer on the surface of AuNRs. Dynamic light scattering (DLS) was used to measure the hydrodynamic size of AuNRs and BSA-coated AuNRs dispersed in water (Figure 1b). The average hydrodynamic size of AuNRs and BSA-coated AuNRs was 55.3 ± 5.8 and 68.1 ± 8.2 nm, respectively. The average hydrodynamic size of BSA-coated AuNRs was a little larger than that of AuNRs, which should be due to the BSA coating layer on the surface of AuNRs. Zeta potential was measured to check the change of surface charge after BSA coating. As shown in Figure 1c, the zeta potential of AuNRs and BSA-coated AuNRs was 41.43 ± 3.80 and −28.82 ± 1.63 mV, respectively, which indicated the surface charge of AuNRs was changed from positive to negative after BSA coating.

**Figure 2:**
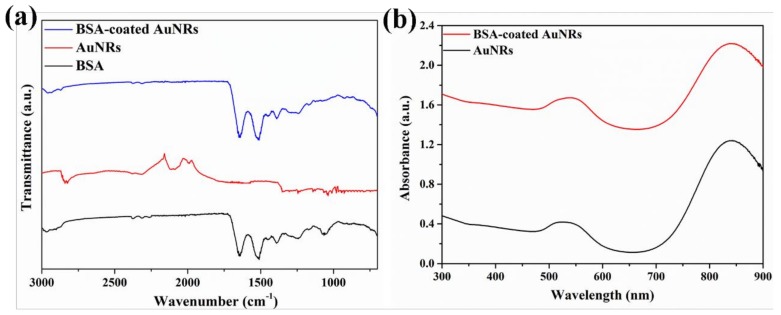
Fourier transform infrared spectra of bovine serum albumin (BSA), AuNRs and BSA-coated AuNRs (**a**) and VIS-NIR spectra of AuNRs and BSA-coated AuNRs (**b**).

Fourier transform infrared spectrum was used to further confirm the successful coating of BSA on the surface of AuNRs. As shown in Figure 2a, the BSA-coated AuNRs displayed two obvious peaks at 1640 and 1524 cm^−1^, which should be attributed to the amide I and amide II bands in the BSA. In contrast, for the naked AuNRs, no peaks were observed at these wavenumbers, which indicated the BSA was successfully coated on the surface of AuNRs. Bovine serum albumin molecules should be bound to the surface of AuNRs through electrostatic interaction of positively charged AuNRs and negatively charged BSA and Au-S bond between AuNRs and thiol groups in BSA. Colloid solutions of BSA-coated AuNRs in ultrapure water and medium were stable without aggregation for a long time, indicating stable BSA coating on the surface of AuNRs. Protein-coating has been reported to increase colloid stability of gold nanorods [39].

A visible-near infrared absorption spectrum (Figure 2b) was used to characterize the optical property of AuNRs. Both AuNRs and BSA-coated AuNRs showed two obvious surface plasmon resonance (SPR) peaks at 535 nm and 848 nm, which corresponded to their transverse and longitudinal plasmon modes, respectively.

### 3.2. Photothermal Performance of BSA-Coated AuNRs

**Figure 3:**
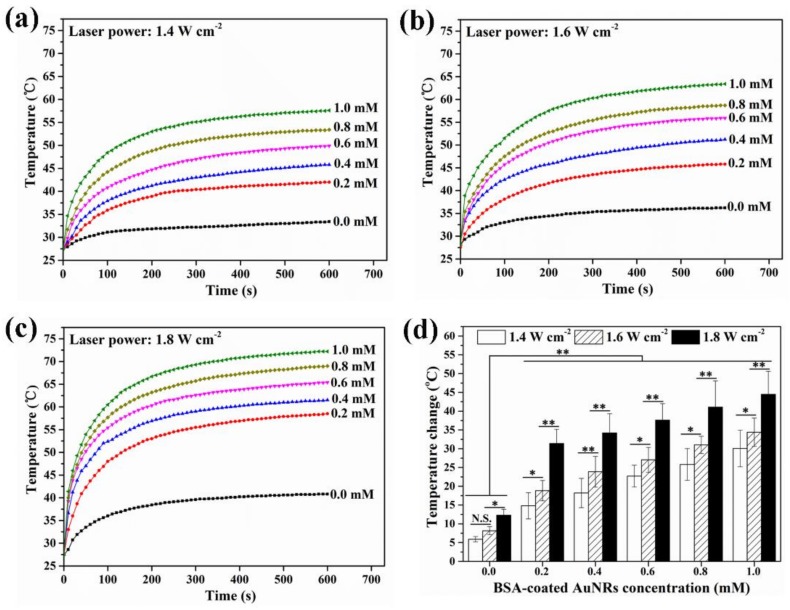
Heating curves of BSA-coated AuNRs aqueous solution at different Au concentration of 0.0, 0.2, 0.4, 0.6, 0.8 and 1.0 mM under continuous NIR irradiation with different laser power intensity of 1.4, 1.6 and 1.8 W cm^−2^ (**a**–**c**). Temperature change of BSA-coated AuNRs aqueous solution at different Au concentration of 0.0, 0.2, 0.4 and 0.6, 0.8 and 1.0 mM after NIR irradiation with different laser power intensity of 1.4, 1.6 and 1.8 W cm^−2^ for 10 min (**d**). Data are presented as mean ± standard deviation, n = 3. No significant difference: N.S.; Significant difference: * *p* < 0.01, ** *p* < 0.01.

Photothermal performance of BSA-coated AuNRs was evaluated by irradiation with an 805 nm laser. Different laser irradiation intensities (1.4, 1.6 and 1.8 W cm^−2^) were used. Heating curves (Figure 3a–c) showed that the temperature of BSA-coated AuNRs aqueous solution in medium increased rapidly compared with control group (0.0 mM, only medium without AuNRs). Temperature increase rate became faster when Au concentration was higher and the laser intensity became stronger. Temperature change data (Figure 3d) after laser irradiation with an intensity of 1.4, 1.6 and 1.8 W cm^−2^ for 10 min indicated that the temperature change of BSA-coated AuNRs aqueous solution increased from 5.9 ± 0.7 to 30.1 ± 4.8 °C, from 8.5 ± 1.1 to 34.4 ± 3.8 °C and from 12.3 ± 1.6 °C to 44.5 ± 6.1 °C, respectively, when the concentration of AuNRs aqueous solution varied from 0.0 mM to 1.0 mM. All these results suggested that the BSA-coated AuNRs had an excellent photothermal conversion efficiency and their photothermal conversion efficiency could be modulated by changing AuNRs concentration in the aqueous solution and laser power intensity. Laser power intensity of 1.6 W cm^−2^ was used in following experiments because temperature increase of BSA-coated AuNRs solution was high while temperature change of control (medium without AuNRs) was not so high.

Bovine serum albumin-coated AuNRs aqueous solution in medium at different Au concentration of 0.0, 0.2, 0.4, 0.6, 0.8 and 1.0 mM was repeatedly irradiated for 5 cycles under continuous NIR irradiation with a laser power intensity of 1.6 W cm^−2^ for 10 min (Figure S1). The heating curves (Figure S1a) were almost the same for each cycle. Visible-near infrared absorption spectrum (Figure S1b) and zeta potential (Figure S1c) of BSA-coated AuNRs did not change before or after laser irradiation. The results suggested photothermal stability of the BSA-coated AuNRs during laser irradiation.

### 3.3. Influence of BSA-Coated AuNRs on Cell Viability

**Figure 4:**
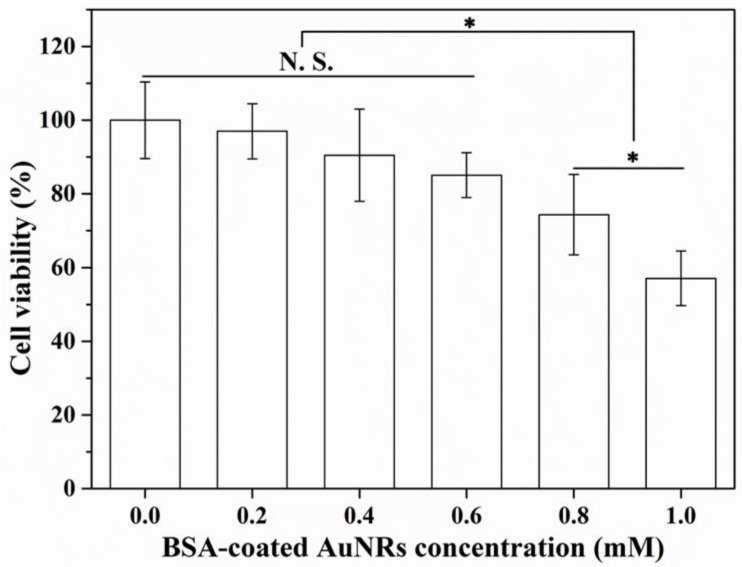
Cell viability of 4T1-Luc cells incubated in medium containing BSA-coated AuNRs at different Au concentrations (0.0 mM, 0.2 mM, 0.4 mM, 0.6 mM, 0.8 mM and 1.0 mM) for 1 day. Data are presented as mean ± standard deviation, n = 3. No significant difference: N.S.; significant difference: * *p* < 0.05.

Cell viability decreased when Au concentration increased (Figure 4). The 4T1-Luc cells cultured in medium containing BSA-AuNRs at an Au concentration of 0.6 mM still showed a high viability (85.0% compared to that of control group). However, when concentration of BSA-AuNRs increased to 0.8 and 1.0 mM, cell viability decreased to 74.4 ± 10.8% and 57.1 ± 7.4%, respectively. For biomedical application, BSA-coated AuNRs as a photothermal conversion agent should have low toxicity to cells before laser irradiation. Cell ablation effect should be switched on only when laser irradiation is applied. Therefore, BSA-coated AuNRs at an Au concentration less than 0.6 mM were used to investigate photothermal ablation efficiency of tumor cells in the following experiment.

### 3.4. Cellular Uptake of BSA-Coated AuNRs

**Figure 5:**
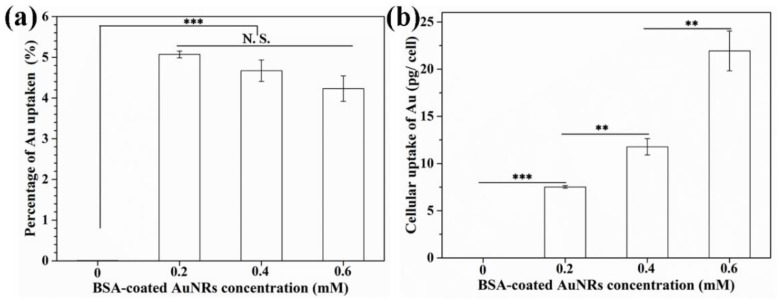
Percentage of Au uptaken by 4T1-Luc cells (**a**) and cellular uptake amount of Au per cell (**b**) after being cultured in medium containing BSA-coated AuNRs at different Au concentration of 0.0, 0.2, 0.4 and 0.6 mM for 6 h. Data are presented as mean ± standard deviation, n = 3. No significant difference: N.S.; Significant difference: ** *p* < 0.01; *** *p* < 0.001.

Cellular uptake amount of BSA-coated AuNRs was measured by ICP-OES (Figure 5). The BSA-coated AuNRs were uptaken during cell culture. After 4T1-Luc cells were cultured in medium containing BSA-coated AuNRs at different Au concentrations of 0.2, 0.4 and 0.6 mM for 6 h, the percentage of Au uptake was 5.1 ± 0.1%, 4.7 ± 0.3% and 4.2 ± 0.3%, respectively, which was not significantly different (Figure 5a). Cellular uptake amount of BSA-coated AuNRs was 7.5 ± 0.1 pg/cell, 11.8 ± 0.9 pg/cell and 21.9 ± 2.1 pg/cell, respectively (Figure 5b). Cellular uptake amount of BSA-coated AuNRs significantly increased when Au concentration in the medium increased.

### 3.5. Photothermal Ablation Effect of BSA-Coated AuNRs

**Figure 6:**
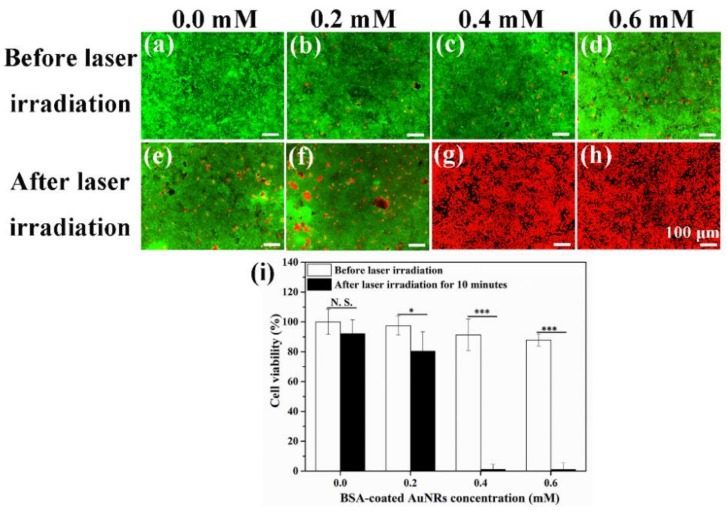
Live/dead staining (**a**–**h**) and viability (**i**) of 4T1-Luc cells before and after NIR laser irradiation for 10 min after the cells were cultured in medium containing BSA-coated AuNRs at different Au concentrations of 0.0, 0.2, 0.4 and 0.6 mM for 6 h. Green color indicates live cells while red color indicates dead cells. Data are presented as mean ± standard deviation, n = 3. No significant difference: N.S.; significant difference: * *p* < 0.05; *** *p* < 0.001.

Photothermal ablation effect of BSA-coated AuNRs was evaluated by using calcein-AM/PI double staining kit and WST-1 assay. Live/dead staining images (Figure 6a–h) showed that after incubation with BSA-coated AuNRs at an Au concentration of 0.0, 0.2, 0.4 and 0.6 mM for 6 h, most of the cells were live before laser irradiation. However, after laser irradiation for 10 min, a small portion of 4T1-Luc cells were dead in the group cultured with an Au concentration of 0.2 mM and almost all the tumor cells were dead in the groups cultured with an Au concentration of 0.4 and 0.6 mM. The cells of control group remained alive even after laser irradiation. In addition, quantification of cell viability (Figure 6i) showed that cell viability in the groups cultured with BSA-coated AuNRs at an Au concentration of 0.0, 0.2, 0.4 and 0.6 mM decreased to 92.1 ± 9.3%, 80.4 ± 13.0%, 1.1 ± 3.3% and 1.0 ± 4.3%, respectively, after laser irradiation for 10 min. The results indicated that photothermal ablation effect of BSA-coated AuNRs increased with the increase of Au concentration. Incubation with BSA-coated AuNRs at an Au concentration of 0.4 and 0.6 mM could ablate all the tumor cells by laser irradiation.

### 3.6. Immune Responses of Immature DCs Triggered by Photothermal Ablated Breast Tumor Cells

**Figure 7:**
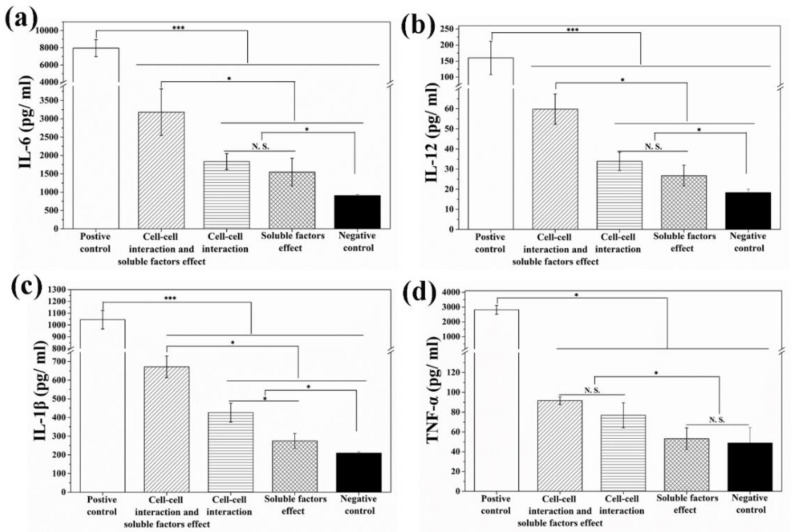
Secretion level of cytokines IL-6 (**a**), IL-12 (**b**), IL-1β (**c**) and TNF-α (**d**) by dendritic cells (DCs) after co-culture of immature DCs with photothermally ablated tumor cells under different co-culture models. Data are presented as mean ± standard deviation, n = 3. No significant difference: N.S.; significant difference: * *p* < 0.05; *** *p* < 0.001.

Effects of soluble factors released from the ablated 4T1-Luc cells, direct cell–cell interaction and their combination on DCs were analyzed by using three different cell-culture models to distinguish these effects. Secretion of multiple cytokines including IL-6, IL-12, IL-1β and TNF-α from DCs was measured by ELISA Kit after transwell co-culture (diffusion model) or direct co-culture of immature DCs with the ablated breast tumor cells (cell contacting model) for 24 h. As shown in Figure 7, both cell–cell interaction and soluble factors released from ablated 4T1-Luc cells promoted the secretion level of IL-6, IL-12 and IL-1β as compared to those of negative control group. As for the secretion of TNF-α (Figure 7d), only cell–cell interaction showed a promotive effect, while soluble factors released from the ablated 4T1-Luc cells could not promote TNF-α secretion as compared with the negative control group.

## 4. Discussion

Photothermal therapy using NIR light-responsive nanoparticles to ablate primary tumor has been widely studied [16,40,41]. However, it remains unclear if the ablated tumor cells can stimulate immune responses. In this study, BSA-coated AuNRs were prepared and used to ablate tumor cells for investigation of the interaction between ablated tumor cells and immune cells. Photothermal ablation efficiency towards breast tumor cells after cellular uptake of BSA-coated AuNRs and immune-stimulatory response of DCs triggered by ablated breast tumor cells were investigated.

Gold nanorods were synthesized by a seed-mediated growth method. They were further coated with BSA for good colloid stability and biocompatibility. The BSA-coated AuNRs had a rod-like morphology and showed a strong SPR adsorption peak at 848 nm corresponding to their longitudinal plasmon modes, which indicated AuNRs could be used as a photothermal conversion agent. The heating curve and temperature change induced by NIR laser irradiation could be modulated by changing the concentration of AuNRs and laser power intensity, which indicated that BSA-coated AuNRs had an excellent photothermal performance. Moreover, heating curves from five cycles of laser on/off suggested BSA-coated AuNRs had excellent photothermal stability.

The BSA-coated AuNRs possessed low cytotoxicity in a broad Au concentration range of 0.0–0.6 mM. In this study, an Au concentration of 0.4 mM was used for photothermal ablation experiments. If gold nanoparticles are hybridized with other matrices, the amount of gold nanoparticles can be further increased [42]. Hybridization of various nanoparticles with polymer matrices has been used to increase biocompatibility of nanoparticles for controlling cell functions [19,38]. In this study, after the breast tumor cells were cultured with BSA-coated AuNRs, BSA-coated AuNRs could be uptaken by tumor cells and the cellular uptake amount increased significantly with the increase of AuNRs concentration in culture medium, which should be beneficial for photothermal ablation. The breast tumor cells could be efficiently ablated by the uptaken BSA-coated AuNRs after NIR laser irradiation when the Au concentration in cell culture medium was 0.4 and 0.6 mM. At a low Au concentration of 0.2 mM, the BSA-coated AuNRs could partially ablate the breast tumor cells. When Au concentration was higher, more AuNRs were uptaken by cells and more rapid heating and higher temperatures could be reached to ablate tumor cells.

To investigate whether the photothermally ablated tumor cells could induce immune response, immature DCs as a key antigen-presenting cells [43] were directly co-cultured with photothermally ablated tumor cells for 24 h. Moreover, to determine whether soluble factors released from photothermally ablated tumor cells were involved in DCs maturation, immature DCs and photothermally ablated tumor cells were co-cultured in a diffusion model, which enabled diffusion of soluble factor in the culture medium while blocking direct cell-to-cell contact. When DCs are matured, they can secrete various types of cytokines to regulate other types of immune cells such as T lymphocyte, which plays crucial roles in inducing the immune responses [44]. IL-6, IL-12 and IL-1β are typical markers of humoral immunity [33] and TNF-α is a typical marker of cellular immunity [33,45]. Secretion of IL-6, IL-12 and IL-1β was promoted by both cell–cell interaction (cell contact) and soluble factors, while secretion of TNF-α was only promoted by cell-cell interaction. The results suggested that photothermally ablated tumor cells by BSA-coated AuNRs were able to induce immune responses of DCs, which should be useful for cancer immunotherapy.

## 5. Conclusions

Gold nanorods were prepared by a seed-mediated growth method and coated with BSA for photothermal ablation of breast tumor cells. The BSA-coated AuNRs showed good monodispersity, low cytotoxicity as well as high cellular uptake by tumor cells in the studied concentration. The BSA-coated AuNRs showed excellent photothermal performance based on the strong SPR absorption in NIR region and showed high photothermal ablating efficiency towards breast tumor cells. Moreover, the photothermally ablated tumor cells triggered immune-stimulatory responses of immature DCs through both cell–cell interaction and soluble factors released from the ablated tumor cells.

## Supplementary Materials

The following are available online at http://www.mdpi.com/1996-1944/12/1/31/s1. Figure S1: Temperature change curves of BSA-coated AuNRs aqueous solution in medium at different Au concentration of 0.0, 0.2, 0.4 and 0.6, 0.8 and 1.0 mM over five laser on-off cycles (a). VIS-NIR spectrum (b) and Zeta potential (c) of BSA-coated AuNRs before laser irradiation and after five laser on-off cycles. The laser power intensity of 1.6 W cm^−2^ was used. Data are presented as mean ± standard deviation, n = 3. No significant difference: N.S.
