# Supplementary material for: Photothermal Ablation of Cancer Cells by Albumin-Modified Gold Nanorods and Activation of Dendritic Cells
Source: Materials (Basel). 2018 Dec 21;12(1):31. doi: 10.3390/ma12010031 (PMC6337519; doi:10.3390/ma12010031)
Supplement: Supplementary file 1 [file materials-12-00031-s001.pdf]

Photothermal Ablation of Cancer Cells by Albumin-Modified Gold Nanorods and Activation of Dendritic Cells

Xiuhui Wang [1,2], Jingchao Li [1,2], Naoki Kawazoe [1] and Guoping Chen [1,2,*]

[1] Research Center for Functional Materials, National Institute for Materials Science,1-1 Namiki, Tsukuba, Ibaraki 305-0044, Japan

[2] Department of Materials Science and Engineering, Graduate School of Pure and Applied Sciences, University of Tsukuba, 1-1-1 Tennodai, Tsukuba, Ibaraki 305-8571, Japan

* Correspondence: Guoping.Chen@nims.go.jp; Tel.: +81-29-860-4496

Photothermal stability of BSA-coated AuNRs

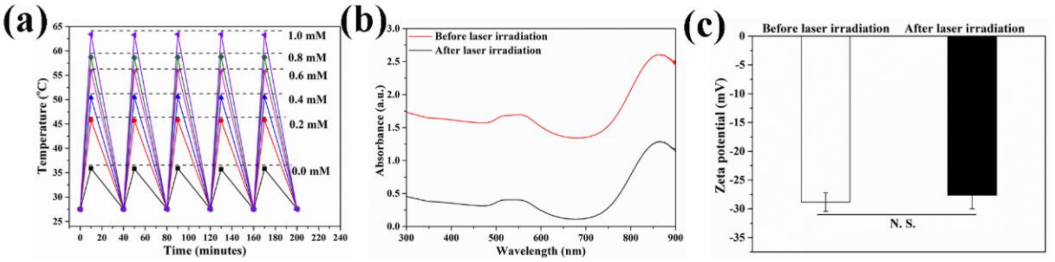

Figure S1. Temperature change curves of BSA-coated AuNRs aqueous solution in medium at different Au concentration of 0.0, 0.2, 0.4 and 0.6, 0.8 and 1.0 mM over five laser on-off cycles (**a**). VIS-NIR spectrum (**b**) and Zeta potential (**c**) of BSA-coated AuNRs before laser irradiation and after five laser on-off cycles. The laser power intensity of 1.6 W cm^{-2} was used. Data are presented as mean ± standard deviation, n = 3. No significant difference: N.S.
